# Sustainable benefits of mindfulness training in health professions education

**DOI:** 10.1186/s12909-025-06998-y

**Published:** 2025-03-27

**Authors:** Camilla Sköld, Anton Steen, Maria Niemi, Bo Vinnars, Anna Kiessling

**Affiliations:** 1 Department of Clinical Sciences Danderyd Hospital, Karolinska Institutet, Stockholm, 182 88 Sweden; 2 Department of Global Public Health, Karolinska Institutet, Stockholm, Sweden; 3 Department of Clinical Neuro Science, Karolinska Institutet, Stockholm, Sweden

**Keywords:** Health professions education, Mindfulness, Self-compassion, Stress, Sustainable

## Abstract

**Background:**

Healthcare work and even studies towards a healthcare profession is associated with a high prevalence of distress. According to recent meta-analyses, half of the medical students worldwide suffer from burn out prior to residency, and 34% of nursing students suffer from depression. The aim of this study was to investigate healthcare students’ long-term experiences of mindfulness training, and whether, how and why students were continuing their mindfulness practice after graduation. Further, to assess if background characteristics were associated with continuing mindfulness practice.

**Methods:**

A mixed method survey study based on predetermined and open-ended questions. Qualitative and quantitative data were analysed concurrently to assess effects on, and a deepened understanding of stress management, use of mindfulness in relation to patients, oneself and others, and continued practice of mindfulness.

**Results:**

Two hundred one of the 380 (52,8%) students completing the mindfulness based stress management course (MBSM) answered the questionnaire. Of all, 175 (87,1%) students also answered free text questions. The qualitative analysis identified five themes: “Positive” Experiences, “Negative” Experiences, “Origin and development of interest in mindfulness” and “Continuing practice of mindfulness”. A considerable proportion of the participants experienced increased acceptance, relaxation, ability to face difficulties, self-compassion and better interaction with patients, but some participants also experienced negative experiences such as aversiveness and ineffectiveness of the course.

Those continuing to practice mindfulness after graduation were more likely (85.3%) to say that the gained competence helped in their relationship with patients, compared to those who did not continue to practice (57.1%); (*chi2* = 18.13; *df* = 2; *p* < 0.001). Among those who had previous mindfulness or similar experience, 84% continue to practice mindfulness after the course, compared to 50% of those who had no previous experience (*chi2* = 26; *df* = 1; *p* < 0.001).

**Conclusion:**

The long-term follow-up of mindfulness training for healthcare students shows that participants maintain a sustained capability to handle stressful work situations in their professional practice and develop a more compassionate relationship with themselves. We argue that skills to care for one's inner environment, such as learning mindfulness as a student, can contribute to a sustainable future professional life. However, further research is needed to confirm the transferability of the results.

**Supplementary Information:**

The online version contains supplementary material available at 10.1186/s12909-025-06998-y.

## Background

Healthcare work is associated with a high prevalence of distress, which is also found among students in healthcare education [1–3]. For example, studies among medical students have shown that they experience higher rates of depression, burnout, anxiety, and suicidal ideation that the general age-matched population [4–6]. Indeed, half of the medical students worldwide suffer from burnout prior to residency, and 34% of nursing students suffer from depression, according to meta-analyses [4]. Moreover, mental health has been found to deteriorate when entering medical school [7–9], and thus it has been postulated that the educational process within the medical programme may be a contributing factor [6].

It is a core skill for healthcare professionals to make complex clinical decisions under the influence of stress and simultaneously being aware of the impact of these decisions on the patient’s health and wellbeing. While doing this, they need to remain empathetic in the caregiver-patient relationship [10, 11]. Yet, the fact that healthcare professionals are working under stress for prolonged periods of time seems to be an increasing problem, resulting in high rates of stress- and work-related burnout [1, 12].

Lately, interventions, such as Mindfulness-Based Stress Reduction (MBSR)[13] and its subsequent offspring of different adaptations called Mindfulness Based Intervention (MBIs), have been receiving an increased research interest for use as stress-management and health-promoting applications in a variety of settings [14, 15]. Several studies have deployed MBSR or other forms of MBIs for healthcare students, to increase their future resilience to the tolls of a healthcare profession, or to increase their empathy and promote their professional development [2, 3, 16–18].

MBSR is based on the core practices of body scan, mindful yoga and sitting meditation. Each group session follows foundational themes such as automatic reactions, perception, pleasant and unpleasant events, stress reactivity and stress response, communication, etc. MBSR is further based on a student activating pedagogic strategy with learning together from peers under simultaneous supervision of a teacher acting as a facilitator and role model. Peer-learning can be defined as “people of similar social groupings who are not professional teachers helping each other to learn” [19]. The method has shown several positive effects from a student and learning perspective. In the context of the present study, the positive effects of peer-learning on self-confidence are particularly interesting [20].

Amongst medical and nursing students, MBIs have been shown to reduce stress levels including perceived stress in relation to exams, improve academic success and increase students’ experience of contentment and wellbeing [2, 21–24].

A recent systematic review of mindfulness-based interventions (mainly MBSR & MBCT) among medical students revealed statistically significant reductions in stress and distress and increased mindfulness in intervention groups compared to controls. These benefits persisted in some follow-up studies (months to years). The effectiveness of MBIs was similar in both shorter and longer courses (4–10 weeks), with or without face-to-face sessions. MBIs show promise for improving medical students' well-being and the study suggests that incorporating them into medical school curricula may be beneficial [25].

MBIs have also been applied successfully among professionals working in clinical settings, where the skills taught have been found to decrease burn out-levels amongst doctors and nurses [12]. Also, these interventions seem to enable healthcare personnel to retain their clinical skills despite the stress invoked by working in the healthcare context [26–29].

A qualitative study among nurses showed that the primary reason to participate in an MBSR intervention was to reduce their own stress levels. Amongst the benefits found in that study were increased relaxation and self-compassion [30]. In another qualitative study with nurses participating in an MBSR intervention, Frisvold, Lindquist and McAlpine [31] found the perceived effects of achieving effective ways to deal with stress, enhanced interpersonal communication, increased self-reflection and awareness of one’s own behaviour as well as spiritual awakening.

At Karolinska Institutet, Stockholm Sweden a Mindfulness-based stress management (MBSM) course has been provided since 2007, where undergraduate students from different study programs towards a multitude of healthcare professions participate together in this interprofessional accredited elective course as part of their curriculum. The course generates 7.5 ECT credits. The course starts with the original MBSR program which is followed by lectures, seminars and assignments. The aim of the course is to obtain knowledge and skills to better cope with stress when working in the healthcare environment as well as to better meet their own and their patients’ emotional needs while concurrently performing better at work.

Taking care of one's own well-being is central to sustainable health, working life and relationships over time. Sweden has taken the initiative and is working together globally to support the so-called Inner Development Goals (IDGs) [32]: According to this initiative, while the United Nations’ Sustainable Development Goals (SDGs) [33] present a comprehensive plan for a sustainable world by 2030, progress toward these goals has not been fast enough, and the inner conditions of individuals seem to be lacking attention in the SDGs. To addresses this gap, the IDGs have been jointly developed, consisting of 5 dimensions with 23 skills for human inner growth and development. The dimensions relate to skills of being and presence, cognitive skills, relationships with oneself and others, cooperation skills and the ability to act.

Previous studies on students have to our knowledge merely investigated the effects of extracurricular implementations of MBIs for students [2, 18]. Outcomes related to learning are regularly evaluated directly after a course, and most studies describing the effects of participation in a MBI have short follow-up times. However, the outcomes related to learning can be perceived differently in a long-term perspective. To our knowledge this study is the only one so far, describing long-term outcomes of an undergraduate intra-curricular MBI course.

The aim of the present study was to investigate the students’ long-term perceptions of obtained knowledge and skills in the course, and whether, how and why students were continuing their mindfulness practice after graduation. A further aim was to assess if some background characteristics were associated with asserted continued mindfulness practice.

## Material and methods

### Design and setting

A mixed method survey study. Qualitative and quantitative data were collected and analysed concurrently. Quantitative data were analysed to assess existence and frequencies of effects on stress management, use of mindfulness in relation to patients, oneself and others, and continued practice of mindfulness. Qualitative data was analysed to deepen the understanding of those aspects.

### Participants

All the 394 students who had participated in the MBSM course during the study period from autumn 2007 to autumn 2015 were asked to participate in the study. During that time frame 27 groups of students attended the MBSM course. Out of these, 14 students did not pass the exam and were thus excluded from participation in the study. When answering the survey, the participants had either graduated or were still students in a study program towards a healthcare profession. Out of the 380 eligible students 201 answered the survey and constituted the basis of the quantitative analyses. Some students received the survey one year after completing the course and others received it at a maximum of nine years after completing the course. The mean time of receiving the survey after completion of the course was 4.3 (SD 2.2) years, and the median time was 5 years. The frequency distribution of the length of follow-up is presented in Table 1.

**Table 1:** Frequence distribution of the length of follow-up between finishing the course and answering of the questionnaire (*n* = 201)

**Length of follow-up (years) **	**n (%)**
1	25 (12.4)
2	28 (13.9)
3	32 (15.9)
4	15 (7.5)
5	34 (16.9)
6	31 (15.4)
7	23 (11.4)
8	8 (4.0)
9	5 (2.5)

Number in brackets denote percentage of the total number of respondents

**Fig. 1:**
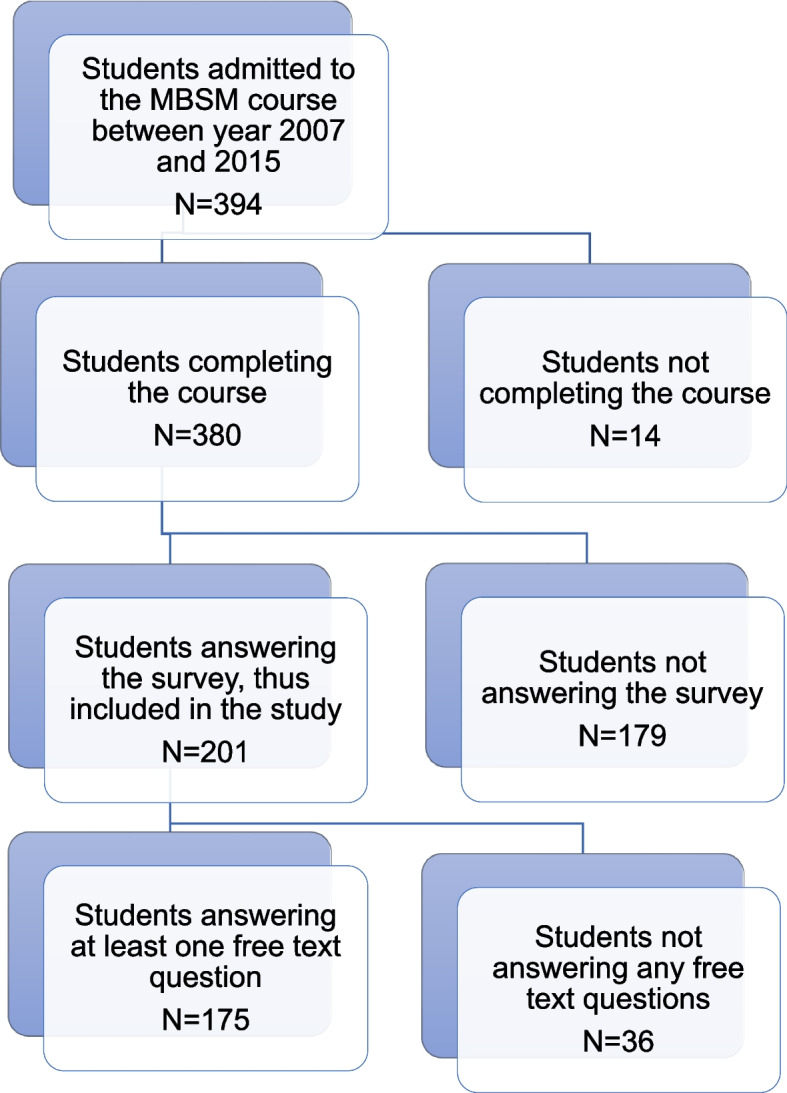
Flowchart of the dropout at various stages of data retrieval

Out of the 201, 175 students also answered one or more of the eleven free text questions and thereby made up the basis for the qualitative dataset of the present study (see Fig. 1).

The students who participated in this study were enrolled in study programmes towards professions as doctor, nurse, psychologist, physiotherapist, midwife, audiologist, speech therapist, biomedical analyst as well as occupational therapist.

### Processes

#### Educational intervention

Since 2007, the MBSM course is still taught every semester and runs at a pace of 25% of full-time education. Between the years of relevance to this present study, 2007 – 2015, the MBSM course has not been mandatory, but available as an optional course for all students studying at a programme towards a healthcare profession at Karolinska Institutet [34, 35].

To facilitate meaningful learning [36] the course has a “backward” design inspired by the experiential learning framework [37, 38] starting with skills training and active student participation before the theoretical and analytical learning activities. The purpose is for students to have their own experience of mindfulness practice as reference, when learning the theory behind it. During the course, students are interacting and learning together in interprofessional groups giving them possibility to learn from their own and others’ reflections on mindfulness and future professional roles.

The course begins with the MBSR programme of eight group sessions of two and a half to three hours each. The students participate in different mindfulness practices, as the body scan, sitting meditation and mindful yoga. Further, they have daily assignments of mindfulness practices and take part in a whole day of mindfulness practices in silence. Each session follows a theme based on the previous week’s home reflection exercise. The second part focuses on theory. The learning activities include participation in practice sessions, lectures, seminars, and assignments. During these, the students attain knowledge of the support for mindfulness-based methodologies, as well as their underlying processes and proposed mechanisms of effect. Examples of topics covered were mindfulness in relation to needs in different groups, physical and psychological symptoms, neuroscientific studies, mind wandering, default mode network etc. The learning objectives, learning activities and examinations are presented in Table 2.

**Table 2:** Constructive alignment mapping of course objectives and forms of examination

**Learning objective**	**Learning activities**	**Examination form**
**Describe how the concept of mindfulness is defined according to international consensus**	Participation and active engagement in the MBSR-programme^a^, self-studies and lectures	Active participation in the MBSR-programme^a^ Oral sharing with peers and teacher/s of experiential understanding of the concept of mindfulness
**Explain how mindfulness is practiced and how this experience and knowledge can be applied in situations of personal mental and physical stress**	Participation and active engagement in the MBSR-programme^a^	Written reflection of own learning process Oral sharing of own learning process in smaller teacher led student groups
**Analyse and reflect on the research and theoretical background on which mindfulness-based methodology rests in relation to own experience-based understanding and knowledge and to explain and justify current research**	Preparatory self-studies, group discussion and preparation of a presentation	Oral group presentation in the whole class about how the theory is related to own experience-based understanding
**Assess the usefulness and relevance of mindfulness-based methodology in complex situations such as healthcare and education**	Self-studies to formulate questions and developed answers based on compulsory course literature and deepened by searched empirical research	Oral exam applying a structured model of discussion according to LSE^b^ and addressing relevance in future professions in healthcare, education etc

^a^Mindfulness Based Stress Reduction (MBSR) programme

^b^London School of Economics (LSE)

Perhaps one of the most important building blocks in the design of the MBSM course is the attitude towards the students, to create a safe space for learning in the course environment. Feeling safe to ask for help and express confusion is linked to better academic outcomes. Students are more likely to seek assistance when needed and to actively participate in learning activities when the level of psychological safety is high [39]. Research clearly shows that higher levels of learning are impossible when our brain has been hijacked by complex and strong emotions, and we feel unsafe [40]. The attitudes in mindfulness help to create e.g. safety, trust, compassion, joy, which have been shown to contribute to learning. Integrating mindfulness practices within a framework of psychological safety can significantly enrich the university classroom experience. By fostering both mindfulness and psychological safety, educators can create an environment that supports academic achievement, emotional well-being, and personal growth. This holistic approach not only benefits students’ learning outcomes but also prepares for the complexities of life beyond the classroom. All parts of this course are permeated by the attitudes of mindfulness.

#### Development of the questionnaire

A questionnaire was originally constructed for quality evaluation purposes to answer questions regarding the usefulness of the MBSM course. It has been developed over the years based on answer patterns. The reliability and validity of the questions were further tested in recurrent dialogues with students. The version of the questionnaire used in this study consisted of a selection of questions from the usual quality assurance questionnaire, and a few extra questions were added to answer the research questions: What perceived gain do students have in a longer perspective from taking part in the course? In what form do some students continue to practice formal mindfulness after completing the course? Why do some students continue to practice formal mindfulness, whilst others stop their practice?

The main domains covered in the questionnaire were: descriptive data; previous mindfulness experience; course quality and gained knowledge and understanding during the course; gained knowledge and understanding in a longer perspective; present mindfulness practice.

The phrasing of the final questions chosen for this study has also been quality checked regarding readability, understanding and relevance by persons regularly practicing mindfulness. The final survey contained 51 questions of which 40 were multiple choice questions and 11 were free text questions rendering qualitative data (See Appendix A).

#### Qualitative data analysis

The free text answers were compiled and sorted by participant-ID, and question number. The process of analysing the data was conducted through six separate phases, according to the thematic analysis method of Braun and Clarke [41]. To identify themes, the data was analysed within an essentialist epistemological paradigm, meaning that the analysis was data driven and that the data was interpreted on a semantic level, as it was written, without looking for latent narration and interpretations [41]. After reading and re-reading the body of text several times and taking notes, parts of the text were highlighted as they were classified in initial codes that were generated. As the body of text was covered by the generated codes, patterns in these were gradually identified and grouped into categories and initial themes. The initial themes were continuously reviewed and given definitions through the progression of the analysis. As definitions emerged, quotations that were representative of these definitions were selected and translated from Swedish to English and the translations were checked by co-authors for triangulation.

#### Quantitative data analysis

How mindfulness was presently practiced within the timeframe when the questionnaire was completed, was covered in several questions. Of particular interest were different aspects of awareness while practicing. Four questions covered aspects such as “are you aware of where you have your attention while practicing?”, “are you aware of your attitude towards yourself and what comes up in your mind?”, “are you aware of your intention while practicing”, “are you aware of how you handle whatever comes up in you while practicing?”. It was considered of interest to explore whether the level of awareness influenced the grade of acceptance and compassionate relationship toward oneself. Further how the level of awareness contributed to skills and attitudes helping to deal with stressful and demanding situations.

#### Statistical analysis

Descriptive statistics were applied on participant characteristics and group comparisons. A linear regression analysis was used to explore how variations in the question “Are you aware of where you have your attention while practicing?” affected the attention in relation towards oneself, and towards stress and demanding situations respectively. In all analyses IBM SPSS Statistics version 27 was used.

## Results

### Participant characteristics

Participant characteristics of the 201 (52,8%) answering individuals are presented in Table 3. Regarding the motive for applying to the MBSM course, 28 (14%) participants indicated stress as the primary motive, 118 (59%) applied due to curiosity, and 27 (13.5%) chose the course for other reasons. The mean age amongst the participants was 35.7 years of age (SD = 7.6), spanning between the youngest individual of 24 years of age to the oldest being 54 years old. Out of the 201 participants there were 162 (80,6%) women and 35 (17,4%) men, while 4 (2,0%) of the participants chose not to disclose their gender. All participants included in any of the data analysis had granted a written, informed consent prior to participation in the survey.

**Table 3:** Participant characteristics (*n* = 201)

**Variable**	**n (%)**
**Mean age**^**a**^ **, years (SD)**	35.7 (7.6)
***Gender***^***b***^	
** Woman**	162 (80.6)
** Man**	35 (17.4)
***Active in professional practice***^*c*^	
** Yes**	161 (80.5)
** No**	38 (19.0)
**Previous experience of mindfulness (yes)**	90 (44.8)
***Study programme***	
** Medical doctor**	43 (21.4)
** Psychologist**	57 (28.4)
** Occupational therapist**	51 (25.4)
** Physiotherapist**	35 (17.4)
** Speech therapist**	11 (5.5)
** Audiologist**	2 (1.0)
** Nurse**	1 (0.5)
** Biomedical analyst**	1 (0.5)

^a^12 (6%) did not provide their age

^b^4 (2%) did not provide their gender

^c^1 (0,5%) did not respond whether they are active in their profession or not

### Qualitative results

Five overarching themes of participants experiences and understanding from taking part in the MBSM course were identified;” Positive Experiences”, “Professional practice”, “Negative Experiences”, “Origin and development of interest” and “Continuing practice” (see Table 4).

**Table 4:** Main overarching themes and their respective subordinate categories

**Theme **	**Categories**
**Positive experience**	Acceptance
	Relaxation
	Facing difficulties
	Self-compassion
**Professional practice**	Work-life
	Recommending the method to patients
	Interacting with patients
**Negative experience**	Aversiveness
	Questioning the course
	Ineffectiveness
**Origin and development of interest**	New skills
	Practicality
	Previous experience
**Continued practice**	Intermittent practice
	Formal practice
	Informal practice

#### Positive experiences

On a wide array of occasions throughout the entire dataset, descriptions of positive experiences constitute a recurring pattern. Hereby, participants describe several ways in which they perceive that they have benefitted from enrolling in the MBSM course. Several participants express feeling glad that they had enrolled in the MBSM course, and that they enjoyed the classes and appreciated the teacher. Some participants also describe that they had liked how the MBSM course differed in structure compared to other courses in their programme.

##### Acceptance

The category acceptance includes descriptions of improved abilities to accept things as they are in the present moment, whilst also knowing that things will be ok later. Participants also described changes in how they relate to their thoughts, feelings, and bodily sensations, as well as feeling less compelled to try to change what is and having a sense of greater tolerance for other people’s ways of being. The citations below illustrate various participant descriptions of acceptance:

*“I observe experiences and try to greet them with acceptance. For example, if I am judgemental about myself - [for example] that [I tell myself that] I am lazy - I go into that feeling, and I just let it be there.” Physiotherapist, 2013.*

*“[I have gained] acceptance of my bodily pain. [I have also] become aware of feelings and thoughts, examining them, and letting them exist! [This implies] allowing the anxiety to speak to the point!” Physician, 2010.*

##### Relaxation

Another category is relaxation, which the participants describe either as the ability to feel calm, or even simply discovering that they can feel calm and soothing feelings in the first place. This category also includes descriptions of an increased ability to unwind during weekdays and feeling peaceful during periods of high demand.

“In everyday life, I can find myself doing the different practices that we learned during the course just to unwind.” Physiotherapist, 2012.

The participants also describe feelings of greater presence and clearer attention both during, and upon completion of the MBSM course. They report that they feel more attentive and present than before, as they notice thoughts, feelings, and bodily sensations and how this makes them more aware of different aspects of the context that they find themselves in. Some participants note how this perceived effect lingers even after they have terminated their formal mindfulness practice, while other participants notice themselves losing this apprehension upon termination of their formal mindfulness practice.

##### Facing difficulties

The third category was facing difficulties. Participants report being better at detecting possible difficulties, and things that may be stress-inducing, as well as having a greater capacity to face stressful, and unpleasant, situations and events. Another aspect of the category “facing difficulties” is, a sense of being capable of observing and identifying their own immediate reactions to things before acting upon them automatically, and thus a greater capacity to choose how they want to act with awareness, rather than automaticity.

*“I have an easier time identifying signs of stress and therefore can to a greater extent “choose” how I cope with such situations. I’m generally more aware of feelings and thoughts, which can sometimes lead to me laughing at myself when I realise how I’m thinking or feeling in a particular situation.” Audiologist, 2015.*

##### Self-compassion

A final category is self-compassion. The participants describe this as being kinder and more understanding toward oneself, as well as being non-judgemental of one’s own experiences, even if these may be aversive at times. The citation below illustrates an example of self-compassion.

*“I try to be fonder of myself, and not to clamp down on myself for being under pressure or feeling stressed.” Psychologist, 2010.*

#### Professional practice

A considerable proportion of participant reports relate to different aspects of their “professional practice” and to how they perceive that the MBSM course has impacted their experience of different aspects of their professional skills and work.

##### Work life

An initial category of this theme is work life. This category is characterized by several participant reports of it being advantageous for them to have knowledge of the method of mindfulness in their line of work. Some of these participants also report it being beneficial for their professional practice to be able to utilize mindfulness in their work situation. Other participants state that mindfulness practice facilitates interacting with patients and co-workers alike. Also, some participants state that they have a greater sense of faith in themselves and in their competence in their professional role as healthcare practitioners. Several participants describe that they can use the exercises from the MBSM course in work situations, for their own sake, to deal with difficulties before and/or after interactions with patients. Some also reveal how they use the exercises together with patients to facilitate their work and increase the quality of the healthcare they provide. The following quotations aim to illustrate aspects of the “work life” category:

*“The [MBSM] course opened up for yet another dimension in my demeanor towards the patient, with [increased] empathy and compassion, as well as observation and self-awareness…” Physiotherapist, 2009.*

*“[Skills developed during the MBSM course] I’m more secure in my role as how I want to be as a physiotherapist, I trust in my own capabilities and dare to try new things even when there’s a risk of failure. I feel that it’s easier to ask coworkers for guidance and support without [me] feeling insecure as a physiotherapist. I allow myself to be engaged with patients and simultaneously I know where my responsibility ends, and I can accept that.” Physiotherapist, 2013.*

##### Interacting with patients

Another category this theme consists of is interacting with patients. This category consists of participants describing a change in how they relate to patients, where some describe that they feel as if they have an increased capacity to understand their patients. They report feeling more present, and as if they have a greater capacity to listen.

*“I always refer [patients] to mindfulness. And I use it [mindfulness practices] myself if I get stressed during the conversation [with the patient].” Psychologist, 2010.*

*“How I can relate to patients where I feel insufficient. How I can try to help patients and their related to approach acceptance and to move on, to be able to prioritize care actions, etcetera.” Occupational therapist, 2010.*

##### Mindfulness as a recommended romplementary treatment

A final category of the theme, recommending the method to patients, is characterized by participants reports that having their own experiences of the method facilitates recommending or referring patients to mindfulness programmes. Some participants state that they after the MBSM course feel that they better can tell when mindfulness interventions fit, and in which cases they are not appropriate.

*“I understand what it is that I’m offering my patients and what this [mindfulness] is all about…” Psychologist, 2013.*

#### Negative experiences

Another theme that emerges is reports of different negative experiences. Within this theme, some participants report experiences of difficulty, distress, and discomfort, as well as other perceived obstacles for engaging in and continuing their mindfulness practice.

##### Aversiveness

One of the categories within this theme is aversiveness, which is characterized by feelings of increased stress related to putting in the time, and the effort that the mindfulness practices of the MBSM course require. Some participants report that they prioritise other activities (ranging from spending time with their family to preferring other coping strategies) rather than the exercises taught in the MBSM course. Another part of this category concerned difficult and unpleasant feelings and sensations that participants experienced during mindfulness practice. Several participants report that these feelings and sensations passed with time and with continued practice, while other participants report that it did not. The citations below illustrate various descriptions of aversiveness:

*“Before the start of the [MBSM] course I expected to reduce the stress associated with my studies, but since the [MBSM] course contained so many different time-consuming exercises, it contributed to an increase in my stress levels instead.” Speech therapist, 2012.*

*“Initially, it was tough to be still for such a long time. [I] often had tormenting thoughts about the numbing sensations [that I felt in my body], and about not being able to cope. But this passed [with practice].” Psychologist, 2012.*

Another part of the category regarded participants reports of setting high standards for themselves regarding being a skilled mindfulness practitioner. They report that the demands from the MBSM course felt like they were forced upon them, and that this became a burden for them in a life that was already filled to the brim with high demands. In several cases, the participants reported that this resulted from being enrolled in additional, overlapping study courses.

##### Questioning the course

Another category that recurs in the data within this theme is questioning the cours**e**, entails the sentiment some participants described of the methods conveyed through the MBSM course being inaccessible. A few participants report that they perceive mindfulness as lacking scientific grounding and rather conveying a lifestyle or something of a more religious nature.

*“It [mindfulness] seemed too much like a lifestyle or a religion…” Physician, 2010.*

*“[I] don’t think that “personal development” is suitable for a [university] course format. A course to me equals obtaining new knowledge, not “gaining new insights”. I felt pathologized.” Psychologist, 2014.*

Some participants also report that they wished to change more practical parts of the MBSM course curriculum, and a few participants state that they would have appreciated more continuing support in their personal practice after the termination of the MBSM course. Some participants also report difficulties with the attitudes and behaviour of other students during the classes.

##### Ineffectiveness

A final category of this theme is ineffectiveness. Some participants report not experiencing any effect, or only limited effects, from having enrolled in the MBSM course. Some list prior experiences, and/or practice of mindfulness as the reason behind this perceived lack of effect, while others describe that they did not experience a need to cope with stressful events. And several participants state that they already use other approaches to better cope with stress.

#### Origin and development of interest in mindfulness

Participants describe how they first found out about the method at hand, and that they could enrol in the MBSM course. Some parts of the data also include expressions of interest in pursuing further training and education in different MBIs for reasons such as personal development and practice as well as to incorporate MBIs in their professional practice.

##### Practicality

A category of this theme was denoted as practicality. Some participants descriptions of enrolling in the course and their reasons for participating and practicing mindfulness as simply due to practicality. Or other technical factors regarding the schedule of the course, and the concurrent courses they had to enrol in through their study programme. For others, the choice was more due to preconceived beliefs regarding the requirements of the course and the amount of effort perceived to be required for the MBSM course.

##### Previous experience

Another category of this theme, previous experience. Consisted of participants that had an antecedent interest in mindfulness practice. Amongst these participants, the nature of this interest varied broadly. For some, it stemmed from previous exposure to and/or experience of MBIs and yoga practices. Others expressed that they hoped the MBSM course would lead them to gain better health—physically and/or mentally.

*“[I enrolled in the MBSM course] for personal reasons, as a way to deal with [my] tinnitus.” Occupational therapist, 2015.*

*“I had already started to build an interest in mindfulness through [reading] books and [practicing] meditation.” Psychologist 2013.*

##### New skill

A final category of this theme, termed new skill, was characterized in the data by participants stating that they enrolled in the MBSM course out of a desire to try out or acquire a new practical skill. That they hoped to gain a tool which could prove to be useful in their future lives, both as practitioners of professions in healthcare and medicine, as in their personal life.

Some participants stated that even though at the time of filling in the survey, they had stopped their mindfulness practice, they were interested in resuming their practice further on. Some report that the course led them to develop increased confidence in themselves, and that it initiated a process of personal development that continued after the course had ended. Other participants voiced their interest in pursuing education to become an instructor of MBIs themselves.

“I’m thinking about taking a new course in mindfulness.” Occupational therapist, 2009.

“I will do it after I’m done with my final course in august; my plans are to focus on refinement of my mindfulness-based competencies.” Physiotherapist, 2014.

#### Continuing practice of mindfulness

##### Intermittent practice

A first category is intermittent practice. In a sizable portion of the data, the participants describe how they have continued their practice of mindfulness after the termination of the MBSM course. Some participants describe that they no longer continue their practice, and yet other participants account that they have stopped their practice in certain time periods but have taken it up again at a later stage in their lives.

##### Formal practice

A category of this theme is formal practice. Among those participants who describe continuing their practice, the descriptions as to how this is done varies. Some report that they continue with formal mindfulness, doing the practises (or similar ones) as they did during the MBSM course. Some participants find it hard to keep up the routine of doing structured practises. Others stress the importance of the structured practices for the maintenance of the effect that they perceive the mindfulness practice grant them. A few participants describe that they have stopped doing most of the formal meditation practises, but that they continue with yoga practices.

*“The [MBSM] course filled an anti-stress-function at the time, thanks to the exercises in the classroom. It was only later, in a very stressful situation, that I rediscovered the material and worked through it again with a very good result.” Physician, 2011.*

*“I meditate several times a week, which helps me cope with a demanding day-to-day life.” Speech therapist, 2009.*

##### Informal practice

Another category of the theme is informal practice. Several participants also report that they practice informal mindfulness, which implies utilising the approach of mindfulness in activities of everyday life, rather than engaging in formal, structured practises. The various situations in which informal mindfulness is used, as listed by the participants include when exercising, parenting, listening to music, gardening, eating food, commuting, and performing a variety of household chores.

*“[I practice informal mindfulness] when I am with my child, when I’m taking a stroll, when I’m riding the subway, and when I’m hanging laundry, etcetera.” Occupational therapist, 2011.*

### Quantitative results

#### Coping with stress and demands

The MBSM course has increased participants' perceived ability to cope with stress and demands. Out of 201 participants, 158 (78.6%) responded that they agree or largely agree that attitudes learned during the course have helped them to manage stress after the course (Table 5).

**Table 5:** How and if learning mindfulness practice have improved the ability to cope with stress and demands

	**Agree to a great extent**	**Agree**	**Don’t agree to a great extent**	**Don’t agree at all**	**Not applicable**	**No response**	**Total**
Mindfulness practice has helped me deal with stress and demands after the course	43 (21,4)	115 (57,2)	35 (17,4)	7 (3,5)	0(0)	1 (0,5)	201 (100)
Mindfulness practice has helped me in relationship to my patients	39 (19,7)	100 (50,5)	31 (15,7)	15 (7,8)	13 (6,6)	3 (1,5)	201 (100)
I gained useful insights about myself	51 (25,4)	104 (51,7)	37 (18,4)	8 (4,0)	0 (0)	1 (0,5)	201 (100)
I developed a more accepting/ compassionate relation to myself	44 (21,9)	108 (53,7)	36 (17,9)	11 (5,5)	0 (0)	2 (1,0)	201 (100)
I gained useful insights of my relationship to other people	38 (18,9)	75 (37,3)	67 (33,3)	20 (10,0)	0 (0)	1 (0,5)	201 (100)

Data are presented as number and percentage n (%)

With respect to the question whether they in retrospect perceived that the course had helped them through a stressful time during the course period, the positive response was slightly less n = 120 (59.7%). Furthermore, it is noted that of the 131 participants who continued with mindfulness practice after completing the course, 112 (86.2%) responded that they pause during stressful or demanding situations and are aware of their breathing. These represent 55.7% of all study participants.

#### Mindfulness in relation to patients

Of the total responses (*n* = 198) to the question about the effect of mindfulness in relation to patients, 13 participants answered Not applicable. Of the remaining 185, 75.1% responded Very true and True that mindfulness has a positive effect in their relationship with patients (applies to both participants who are active and not active in their profession) (Table 5).

In further analysis, those who continue to practice mindfulness after the course were more likely (85.3%) to say that the course helps in their relationship with patients, compared to those who do not continue to practice (57.1%). The difference is statistically significant, (*chi-2* = 18.13; *df* = 2; *p* < 0.001).

#### Mindfulness in relation to oneself and others

Regarding the impact of the course on the respondent’s relation towards oneself, 77.1% responded that they gained valuable insights about themselves, 56.2% that they gained valuable insights about their relationship with others (work colleagues, private life) and 75.6% that they developed a more accepting and compassionate relationship with themselves (Table 5).

Furthermore, 120 out of 201 participants (59.7%) felt that learned mindfulness skills and attitudes were helpful in the context of close relationships in their personal lives.

#### Attention

Attention, which is practiced in mindfulness was, applying linear regression analysis, significantly related to how to deal with stressful and demanding situations (*B* = −0.220, *t* = −2.491, *p* = 0.014). However, a linear regression analysis between attention and relation towards oneself showed no significant result (Table 6).

**Table 6:** Linear regression analysis showing the relationship between attention applied in mindfulness, and the independent variables stress and demands, respectively relation towards oneself

**Relationship **	**Beta**	**SE**	**t**	**Sig**
Attention – Relation towards oneself	-.146	.103	−1.622	.107
Attention – Stress and demanding situations	-.220	.080	−2.491	***.014***

#### Continued practice of mindfulness

Analysis showed that of those who had previous mindfulness practice or similar, 84% continue to practice mindfulness after the course, compared to 50% of those who had no previous experience. This difference is statistically significant (*chi-2* = 26; *df* = 1; *p* < 0.001).

Of the participants who failed to maintain practice, 51.2% said it was very likely or likely that they would take it up later in life or when needed.

## Discussion

In the present study the experiences of students going through the MBSM course at Karolinska Institutet have been explored in a long-term perspective. This makes this investigation rather unique and offers an important opportunity to contribute to the field of MBI research and its potential for professional development and future health among healthcare students. The findings illustrate several positive experiences of the MBSM course, but there is variety in the descriptions, where some of them are also negative.

### Coping with stress and demands – positive and negative experiences

The findings of the present study regarding experiencing positive outcomes of the course are compatible with those of previous research showing positive experiences of MBIs conducted among healthcare practitioners [27–29], and healthcare faculty and residency students [27, 42].

Amongst those who perceived negative outcomes from the MBSM course, these were characterized by the experience of distress and other adverse feelings and thoughts, for some during the mindfulness practices and for others due to the demands of the course. Other negative experiences included a lack of effect experienced from performing the practices as well as negative viewpoints on the content or other practical aspects of the course.

The perceived negative experiences align well with results from previous qualitative work [26, 30]. It is noteworthy, that a previous study has found that the sense of a spiritual awakening was amongst the perceived positive outcomes of partaking in an MBI programme [31], while for the results of this present study the perception of religiosity in the MBSM course was instead framed as a negative experience. It should be noted here that these two studies were conducted among different study populations (healthcare students in the present study and healthcare practitioners in midlife for the other study) and thus the differing results might be due to differences in the characteristics of the study populations and non-viable transferability between them [31].

Through employing a longitudinal qualitative study design Kerr et al. [43] have been able to show how negative experiences of performing mindfulness practises don’t necessarily have to imply that participation on a MBI course results in a lack of perceived positive outcomes. On the contrary, they found that perceived positive outcomes can coexist with negative experiences of the practices and that these experiences may change in nature over time [43]. This is of interest in relation to the interpretation of the perceived experiences and outcomes of the present study. Especially since a few participants describe such a shift from perceived distressing sensations initially during practice, which then allegedly passes with time and continued mindfulness practice. It could also imply that even if distressing experiences during the practices were to linger, they might not necessarily prevent the possibility of eventual positive outcomes. But it should be noted that there may be crucial differences between the study populations of healthcare practitioners and healthcare students which limits transferability between the populations and hampers certainty in such interpretations of results.

### Mindfulness in professional practice

The results of the present study are interesting considering research which has shown that healthcare practitioners are often overstrained and that this is a factor contributing to lower quality healthcare. This result of the present study is in line with previous studies which have shown that MBIs may help to alleviate some of these burdens experienced by healthcare practitioners as well as possibly bolstering certain occupational skills [2, 10, 11, 26, 27, 29]. Due in part to methodological restraints, even though these results are promising, they do not warrant application of MBIs to every single healthcare practitioner-to-be, as for that, future research is still needed.

The qualitative findings from this study are to a great extent in line with previous results from qualitative studies amongst both healthcare students and healthcare practitioners, and this seems to be the case both regarding the themes of positive experience and of professional practice [27, 29–31, 42, 44].

### Background characteristics and continued practice of mindfulness

Some participants enrolled in the course due to convenience or other practical reasons, others did so due to their previous experience of MBIs and some did so out of curiosity after hearing of the method, but without having had previous experience. Some participants describe that they participated in the course due to a desire to develop a new set of skills to prepare them for their future profession and/or other challenges of life.

The results indicated that two of the investigated background characteristics, namely applying because of stress and having had previous experiences of MBIs, increased the later likelihood of continued mindfulness practice after the termination of the MBSM course. Nevertheless 50% of those who had no previous experience also continued formal or informal mindfulness practice. The association between applying to cope with one’s own stress and a continued mindfulness practice is in accordance with previous research [44]. Many participants also experienced an impact of the course on work skills and their subsequent professional practice. These descriptions included the ability to utilise the practices and skills acquired from the course to handle various work situations, to better interact with patients and to better distinguish which patients might benefit form mindfulness practice.

Many participants describe a plethora of ways in which they have continued their mindfulness practice after termination of the MBSM course. This occurs through both formal and informal mindfulness. Some find it hard to keep up with the routine of practicing formal mindfulness, and others only continue with a few of the formal practices. Some participants who have stopped their mindfulness practice in certain time periods, state that they have been able to find their way back to them when life circumstances have given rise to a need for it. A few participants state that after finishing the MBSM course they now wish to obtain further education within the field, or that they, despite having stopped their practice of mindfulness practises/skills at the time being, seek to begin once more in the future.

### Strengths and limitations

Previous research shows predominating improvements and positive results of participation in MBI programmes [2, 23]. However, the present study also shows some negative results. This might be due to differences in methodology and in time frame for data collection compared to time for participation in the course. Most other studies of the effects of MBIs in health profession students have utilised quantitative methods and usually have shorter follow-up, which makes comparison to this mixed method long-term follow-up study difficult. However, the difference in effects between our study and other studies seems to remain even in comparison to the study by Oman and colleagues applying a qualitative method [22].

Moreover, the transferability of the present study is possibly hampered by selection bias regarding mix of participants in the course. Most studies on the effects of MBI programmes have investigated elective extra-curricular course participants wile this study investigates previous participants in an elective part of study programmes. This might contribute to the participation of students who would otherwise not go through an MBI programme will do so.

It is a limitation that the questionnaire has not been validated. However, it has been used for course quality evaluation purposes and been developed over the years based on answer patterns and comments from students. The sample of participants for this present study is a convenience sample limited to students enrolled at a study programme towards a healthcare profession at a medical university in Sweden. This may limit the possibilities to achieve representation of the results for other populations of students and/or healthcare practitioners from other cultural and ethnical backgrounds. However, the last twenty years in Sweden have been characterised by a shift towards a multicultural mix of students at the universities. Regardless of this, the results may well represent perspectives of those, among students enrolled at Karolinska Institutet in Sweden, who have chosen the Mindfulness-Based Stress Management course. Also, the response rate for the quantitative data was moderate (201 out of 380) and lower for the qualitative data (179 out of 380). We failed to show significant relation between attention and self-relation. However, the qualitative results indicates that attention to e.g. self-compassion increased. However, no validity issues need to invoke on the present study’s ability to achieve qualitative representativeness. It should be noted that all former participants were contacted, up to 9 years after completing the course. With that in mind, it is a strength that 53% of previous participants did prioritize to take their time to answer.

We believe that a convenience sampling, i.e. asking all participants, is the most appropriate method to evaluation the effects of a course. All educational interventions are context and participant dependent implying that the results never can be generalized but can instead be transferred and adjusted to other contexts. However, the fact that students from so many health professions and cultural backgrounds were included helps to support the transferability to a broader context.

### Implications for future clinical work and research

According to the United Nations [33], sustainable working life should be promoted, and this should be achieved through the strengthening of decent work environments and organizations. We argue that participation in a MBSM course for students has potential to contribute to this goal by improved understanding and training of the IDGs [32], which have been developed to support reaching the SDGs.

It has been shown that people who practice compassionate presence and provide care to others are less likely to become exhausted or experience empathy fatigue [45] and Grepmair and colleagues [46] have found that learning to practice meditation in psychotherapist training can positively influence the therapeutic course and treatment results in their patients. Therefore, mindfulness practice is likely to create a positive ripple effect of improved caregiver wellbeing that in its turn may have a positive impact on patient care.

## Conclusion

An optional interprofessional intra-curricular course, such as the MBSM course for healthcare professional students, incorporating the experiential learning of a mindfulness practice is shown to generate perceived positive outcomes. The long-term follow-up shows promising results where participants feeling able to cope with various stressful work situations and developed a more compassionate relation to oneself once they finish their education and begin their professional practice. Background characteristics associated with continued mindfulness practice include having applied for the course because of stress or having had previous experiences of MBIs. We argue that skills to take care of one’s own inner environment, such as learning of a mindfulness practice as a student, has potential to contribute to a sustainable future professional life. However, further research is necessary to confirm the transferability of the results.

## Supplementary Information

Supplementary Material 1.

## Data Availability

The datasets used and/or analyzed during the current study are available from the corresponding author upon reasonable request.
