# Supplementary material for: Sustainable benefits of mindfulness training in health professions education
Source: BMC Med Educ. 2025 Mar 27;25:451. doi: 10.1186/s12909-025-06998-y (PMC11951767; doi:10.1186/s12909-025-06998-y)
Supplement: Supplementary file 1 — Supplementary Material 1. [file 12909_2025_6998_MOESM1_ESM.docx]

# Course evaluation of the MBI-course (7,5 hp)

(Answered by participants between autumn 2007 and autumn 2015)

**I Demografic data**

1. When did you participate in the course?

Year

⬜ Autumn

⬜ Spring

2. Gender

 Woman

 Man

 Do not want to identify me

3. Year of birth__________

4. What study programme were you admitted to when you started the course?_____________________

5. Why did you apply to the course?

 Stress

 Curiosity

 Other reasons, what? _____________

*6. Number of children at home beneath 18 year ______*

7. Are you today working in the profession you studied during the course?

 Yes

 No

**II Previous experience of mindfulness**

8. Did you have any experience of practising mindfulness or similar activities before the course?

⬜ Yes

⬜ No

If Yes, what?

 Mindfulness

⬜ Vipassana

⬜ Zen

⬜ Transcendental Meditation (TM)

⬜ Christian deep meditation

⬜ Yoga

⬜ Qi-Gong

⬜ Tai Chi

⬜ Feldenkrais

⬜ Other, what?

*9. If you still practice any of the mindfulness forms, how long have you been practicing?*

⬜ < 1 year

⬜ 1-2 year

⬜ 3-5 year

⬜ 5-10 year

⬜ > 10 year, How many years?

10. Had you participated in any form of silent meditation or/and yoga-retreat before the course?

⬜ Yes, wich year?

⬜ No

**III Summary assessment of the course**

*11.* Spontaneously, how would you assess the value of the course?

Mark you assessment on a scale from *1= No value at all* to *10=Very big value? ___________*

**IV Assessment during the course**

Reflect back to the time when you went through the course and answer the questions as best you can from your memories. How accurate is the statement that:

12. The course helped me through a stressful time.

⬜ Very accurate/true

⬜ Accurate

⬜ Not very accurate

⬜ Not accurate at all

⬜ Not applicable

13. During the course, I gained valuable insights into myself.

⬜ Very accurate/true

⬜ Accurate

⬜ Not very accurate

⬜ Not accurate at all

⬜ Not applicable

14. During the course, I gained valuable insights about my relationship with others.

⬜ Very accurate/true

⬜ Accurate

⬜ Not very accurate

⬜ Not accurate at all

⬜ Not applicable

15. During the course it became clearer to me why I chose the education I did.

⬜ Very accurate/true

⬜ Accurate

⬜ Not very accurate

⬜ Not accurate at all

⬜ Not applicable

16. During the course it became clearer to me how I wanted my future professional life to develop.

⬜ Very accurate/true

⬜ Accurate

⬜ Not very accurate

⬜ Not accurate at all

⬜ Not applicable

17. The course was a start to (or an important part of) addressing problems I felt I had in my life.

⬜ Very accurate/true

⬜ Accurate

⬜ Not very accurate

⬜ Not accurate at all

⬜ Not applicable

18. During the course I developed a more accepting and compassionate relationship with myself.

⬜ Very accurate/true

⬜ Accurate

⬜ Not very accurate

⬜ Not accurate at all

⬜ Not applicable

19. The course was a start to (or an important part of) continued active personal development.

⬜ Very accurate/true

⬜ Accurate

⬜ Not very accurate

⬜ Not accurate at all

⬜ Not applicable

20. Please tell us how you experienced the course time, either in relation to the above questions (12-19) or based on your own thoughts and feelings:

**III Assessment of the course after the course until today**

Think about the benefits you might have today from what you experienced and learned during the course. How accurate are the statements:

21. Skills or approaches I developed during the course help me deal with situations that are stressful or otherwise demanding.

⬜ Very accurate/true

⬜ Accurate

⬜ Not very accurate

⬜ Not accurate at all

⬜ Not applicable

22. Skills or approaches I developed during the course help me in close relationships in my private life.

⬜ Very accurate/true

⬜ Accurate

⬜ Not very accurate

⬜ Not accurate at all

⬜ Not applicable

23. Skills or approaches I developed during the course help me in my relationship with patients (or equivalent).

⬜ Very accurate/true

⬜ Accurate

⬜ Not very accurate

⬜ Not accurate at all

⬜ Not applicable

*If so, please tell us how __________________________________________________*

*_________________________________________________________________________________________*

24. Skills or approaches I developed during the course help me in my relationship with colleagues (or equivalent).

⬜ Very accurate/true

⬜ Accurate

⬜ Not very accurate

⬜ Not accurate at all

⬜ Not applicable

25. In my professional practice, I try to help patients (or equivalent) to develop skills or approaches that I myself came into contact with or developed during the course.

⬜ Very accurate/true

⬜ Accurate

⬜ Not very accurate

⬜ Not accurate at all

⬜ Not applicable

26. When dealing with patients (or equivalent), I sometimes use exercises I learned during the course.

⬜ Very accurate/true

⬜ Accurate

⬜ Not very accurate

⬜ Not accurate at all

⬜ Not applicable

27. I refer patients (or equivalent) to mindfulness-based interventions.

⬜ Never

⬜ Rarely

⬜ Sometimes

⬜ Often

⬜ Always

⬜ Not applicable

28. Knowledge or experience from the course is useful in assessing statements about mindfulness.

⬜ Very accurate/true

⬜ Accurate

⬜ Not very accurate

⬜ Not accurate at all

⬜ Not applicable

29. I have continued training in mindfulness or related methodologies to apply in my professional practice.

⬜ Yes

⬜ No

30. If yes, which one?

⬜ MBSR – Mindfulness Based Stress Reduction

⬜ MBCT – Mindfulness Based Cognitive Therapy

⬜ MBRP – Mindfulness Based Relaps Prevention

⬜ ACT – Acceptance and Commitment Therapy

⬜ DBT – Dialectical Behavioral Therapy

⬜  *CFT – Compassion Focused Therapy*

⬜  *MSC – Mindful Self Compassion*

⬜ Other, which one?

31. Please comment on how you feel you have benefited from the course (today), either in relation to the above questions (21-30), or based on your own thoughts and feelings:

**IV Assessment of your practicing today**

32. Have you continued to practice mindfulness, *formally and/or informally*, since completing the *course*?

⬜ Yes

⬜ No

If you answer NO go to question 50, if you answer YES go to question 33.

33. How often do you practice formal mindfulness (body scan, sitting meditation, mindful yoga)?

⬜ Less than once a month

⬜ A few times a month

⬜ A few times a week

⬜ Daily

⬜ Several times a day

⬜ Not applicable

34. On average, how long do you practice *formal mindfulness* on each occasion?

⬜ 1-10 min

⬜ 11-20 min

⬜ 21-30 min

⬜ 31-45 min

⬜ 46-60 min

⬜ >60 min

⬜ Not applicable

35. Are you aware of where your attention is when practicing formal mindfulness?

⬜ Never *or very rarely*

⬜ Rarely

⬜ Sometimes

⬜ Often

⬜ *Very often or* always

36. Are you aware of your attitude (mindset) towards yourself and what emerges during your formal mindfulness practice?

⬜ Never or very rarely

⬜ Rarely

⬜ Sometimes

⬜ Often

⬜ Very often or always

37. Are you aware of your intention when practicing formal mindfulness?

⬜ Never or very rarely

⬜ Rarely

⬜ Sometimes

⬜ Often

⬜ Very often or always

38. Are you aware of how you handle what you notice in yourself when practicing formal mindfulness?

⬜ Never or very rarely

⬜ Rarely

⬜ Sometimes

⬜ Often

⬜ Very often or always

39. Do *you ever practice informal mindfulness, e.g. paying attention to things you do in your daily life*?

⬜ Yes

⬜ No

40. If YES, how often?

⬜ Less than once a month

⬜ A few times per month

⬜ A few times a week

⬜ Daily

⬜ Several times a day

41. Give three examples of everyday situations in which you practice informal mindfulness.

42. Do you use the 3-minute mini-meditation called *"breathing space" or the "hourglass"?*

⬜ Yes

⬜ No

43. If YES, how often?

⬜ Less than once a month

⬜ A few times per month

⬜ A few times a week

⬜ Daily

⬜ Several times a day

44. Give three examples of situations where you use the mini-meditation?

45. Do you stop and be aware of your breathing under stress or in other demanding situations?

⬜ Yes

⬜ No

46. If YES, are you aware of the attitude towards yourself and what is happening?

⬜ Yes

⬜ No

47. Feel free to give examples of how you respond to what you notice about your attitude.

48. How would you rate the value of your continued mindfulness practice - *on a scale of 1-10, where 1 = no value at all and 10 = very high value*?*___________*

49. Please tell us how you practice in your daily life today, either in relation to the questions (32-48), or from your own thoughts and feelings:

*If you answered NO to question 32, continue here:*

50. How likely is it that you will start a mindfulness practice again?

⬜ Very accurate/true

⬜ Accurate

⬜ Not very accurate

⬜ Not accurate at all

⬜ Not applicable

51. Please share why you never got started or stopped practicing mindfulness, and how you see a possible future practice?

**Thank you for participating!**
